# Parity Space-Based Fault Detection for Networked Control Systems Under DoS Attacks

**DOI:** 10.3390/s26134023

**Published:** 2026-06-24

**Authors:** Yang Song, Wenge Sun, Xiaoyu Zhang, Rong Guo

**Affiliations:** 1 School of Intelligent Science and Technology, Beijing University of Civil Engineering and Architecture, Beijing 100044, China; 2108540625019@stu.bucea.edu.cn (W.S.); zhangxiaoyu@bucea.edu.cn (X.Z.); guorong@bucea.edu.cn (R.G.); 2 Beijing Key Laboratory of Robot Bionics and Function Research, Beijing University of Civil Engineering and Architecture, Beijing 100044, China

**Keywords:** parity space, fault detection (FD), networked control systems (NCSs), denial-of-service (DoS) attacks

## Abstract

**Highlights:**

**What are the main findings?**

- The paper constructs a parity relation with a time-varying window for networked control systems (NCSs) under Denial-of-Service (DoS) attacks. This relation is used to develop a residual generator that can prevent transmission errors caused by DoS attacks.
- An adaptive evaluation function with a unified threshold is proposed for residual evaluation. This decision logic is effective in various scenarios, including those without DoS attacks and those with different types of DoS attacks.

**What are the implications of the main findings?**

- The proposed FD method improves the detection performance for NCSs specifically under DoS attacks in the parity space framework.
- The unified threshold and adaptive evaluation allow the same system to operate effectively under both normal and DoS attack conditions, simplifying monitoring logic while maintaining performance.

**Abstract:**

This paper presents a parity space-based fault detection (FD) method for networked control systems (NCSs) subject to denial-of-service (DoS) attacks resulting in packet dropouts. To begin with, a parity relation that takes into account DoS attacks is constructed utilizing the available input and output data during a time-varying window. Based on the parity relation, a residual generator is proposed without introducing transmission errors caused by DoS attacks. Additionally, an $H_{-}/H_{2}$ optimization scheme is developed for the design of residual to achieve a trade-off between the sensitivity to faults and the robustness against the disturbance/noise. Then, an adaptive residual evaluation function with a unified threshold is also proposed for no DoS attack scenario and DoS attack scenarios. Furthermore, the relationship between the traditional residual generator with the proposed one is explored, and an online FD algorithm is presented. Finally, the effectiveness of the proposed approach is demonstrated on a linearized longitudinal model of an autonomous underwater vehicle (AUV), and a comparison with an existing parity space approach is also provided.

## 1. Introduction

Due to the lower cost and more flexible control structures, networked control systems (NCSs) have received significant attention and have been widely applied in various fields, such as unmanned aerial vehicles (UAVs) and autonomous underwater vehicles (AUVs) [1,2,3]. In NCSs, data is transmitted among different component units through communication networks.

A significant amount of effort has been dedicated to addressing fault detection (FD) problems in order to enhance the safety and reliability of NCSs [4,5,6]. Some approaches to FD in NCSs utilize deep learning methods, which employ various networks to extract fault information [7,8]. For instance, a fault classification scheme has been proposed using a hierarchical rule strategy, where the antecedent attributes are extracted from the characteristics of the measurement [7]. Furthermore, a fault diagnosis framework based on a graph neural network has been developed for train transmission systems. This method effectively captures the interaction between components and has improved the diagnosis performance [8]. However, these methods lack physical significance and may yield poor results if the training data is insufficient.

Compared to deep learning techniques, model-based FD methods are characterized by their clear physical meaning, making them more applicable for systems with insufficient historical data. For the FD problem in linear networked Metzler systems, a Luenberger observer has been designed for fault detection filters (FDFs) [9]. Additionally, a distributed observer has been used for state estimation of the leader, allowing for time-varying formation tracking of UAV systems even under actuator faults [10]. However, these approaches do not take into consideration denial-of-service (DoS) attacks on the communication network. These attacks, which have been observed in NCSs [11,12,13], present new challenges in dealing with FD problems with incomplete data.

When considering DoS attacks, numerous studies on NCSs utilize observers/filters to implement secure state estimation or fault-tolerant control in the events of DoS attacks [14,15,16,17,18]. To achieve secure state estimation, an adaptive strategy has been developed that utilizes a switching rule between an initial running observer, a fixed-time observer, and a real-time observer to handle DoS attacks [14]. This approach ensures that state estimation errors converge to a bounded set within a predefined fixed time. Furthermore, a state estimator based on a neural network (NN) has been developed, incorporating an adaptive tuning law for the network weights [15]. The necessary conditions for this estimator have been derived using Lyapunov theory, ensuring the state estimation errors ultimately bounded in mean square under the worst-case DoS attack scenario. For multi-agent systems facing DoS attacks, a distributed secure state estimation scheme has been proposed by using the relative measurement outputs between neighboring agents [16]. Among the various secure state estimation techniques, resilient observers have shown promising results in robust control [17,18]. For instance, an iterative observer design scheme has been devised to estimate both the system state and the attacks for the distributed resilient control of energy storage systems of islanded microgrids [17]. Using the local measurements with neighboring agents, distributed resilient interval observers have been designed for multi-agent systems subject to cyber-attacks, and a four-step recursive distributed algorithm has been developed [18,19].

The above-mentioned state estimation methods provide valuable information despite the DoS attacks, which provide key information for robust control. An adaptive event-triggered control method has been proposed that utilizes a sliding mode controller to defend against security threats in NCSs that are vulnerable to stochastic DoS attacks [20]. Additionally, a fuzzy robust control method has been suggested that utilizes performance recovery analysis for networked systems through a deferred actuator-switching approach [21]. These results provide effective robust control schemes for NCSs subject to controller-to- actuators DoS attacks.

To achieve attack-resilient FD under DoS attacks, a switched-type fault detection filter in a resilient dynamic event-triggered transmission has been proposed to reduce network bandwidth usage while maintaining detection performance [22]. Additionally, a unified resilient observer has been designed using local performance indices for collaborative detection of faults and attacks in cyber-physical systems [23]. For multiple unmanned aerial vehicles (UAVs) subject to DoS attacks, a fixed-time link weight estimation scheme has been designed to reconstruct the neighbor states and actuator faults [24].

In addition to observers/filters-based approaches, parity space-based FD methods, which rely on the parity relation among input/output data during a specific time window, offer a more straightforward form and the ability to decouple residuals from the state vector. The advantages of using parity space methods in NCSs make it a suitable approach for handling incomplete data and improving FD performance. Previous research has explored the use of parity space for detecting replay attacks [25]. However, the challenge of handling missing data caused by DoS attacks has not been given enough attention in the parity space scheme. The traditional parity relation relies on a fixed-length time window of consecutive output samples. However, in the case of a DoS attack causing packet dropout, the straightforward solution is to replace the missing output with the last available value [26]. This substitution breaks the original parity relation because the replaced value does not satisfy the system dynamics. As a result, the residual becomes coupled with transmission errors, which cannot be distinguished from actual faults. This approach often results in existing methods being plagued by either false alarms (if the threshold is set too low) or missed detections (if the threshold is raised to tolerate errors). Therefore, both the parity space-based residual generation and evaluation for NCSs with DoS attacks remain a challenging topic.

Motivated by the observations mentioned above, this paper focuses on addressing the FD problems in NCSs with DoS attacks using a parity space-based approach. Instead of using the traditional parity relation over a fixed time window, we reconstruct the parity relation over a time-varying window that accurately reflects the actual arrival times of available data during DoS attacks. This allows for the development of a parity space-based residual generator that is decoupled from transmission errors, as well as an adaptive residual evaluation scheme. The key contributions of this study are as follows:

(1) The common FD scheme often results in a transmission error when attempting to replace lost data, which can limit detection performance. To address this issue, this paper proposes a solution that involves constructing a parity equation over a time-varying window, determined by the actual arrival times of output data during DoS attacks. This approach completely avoids the transmission errors caused by such attacks.
(2) Based on the parity equation, this paper designs a residual generator using only the inputs and outputs that have successfully arrived at the FD unit. To further improve the performance of the residual generator, an $H_{-}/H_{2}$ optimization problem is proposed for the residual generator to achieve a balance between sensitivity to faults and robustness against disturbances/noise. This approach completely decouples the residual from any transmission errors, eliminating the need for heuristic threshold adjustments.
(3) This paper presents an adaptive evaluation function that adjusts to the varying window length, improving efficiency in both attack-free and attack scenarios. This FD decision logic, which simplifies practical implementation, uses a unified threshold to enhance accuracy.

This paper assumes that the NCS can be described by a linear time-invariant (LTI) model. However, many real-world systems exhibit nonlinear or time-varying dynamics. The proposed method is well-suited for resource-constrained FD units, but it may not be applicable to all systems. Additionally, this work specifically addresses DoS attacks that cause packet losses or repetitions. However, it is important to note that there are other types of attacks, such as replay attacks and stealthy injection attacks, which primarily violate the integrity and authenticity of data. The core contribution of this work is the construction of a parity relation over a time-varying window that adapts to DoS-induced packet losses. It is important to note that there is potential for further research in extending this method to linear parameter-varying or nonlinear models with other attacks.

## 2. Problem Formulation

**Figure 1:**
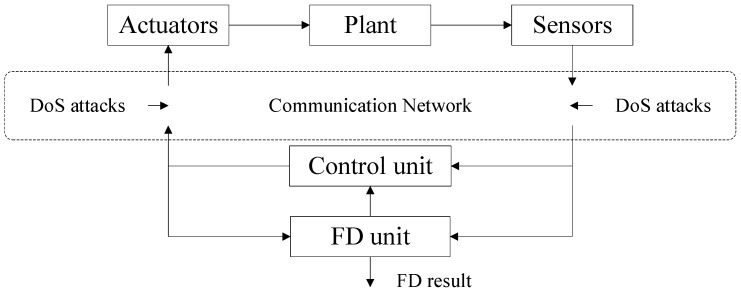
The structure of an NCS subject to DoS attacks.

Consider a NCS consisting of a discrete-time LTI process, actuators, sensors, a control unit, an FD unit and communication networks vulnerable to DoS attacks. The structure of this NCS is illustrated in Figure 1.

The LTI process without any DoS attack can be described as follows:

(1) $$\left\{\begin{array}{l} x(k+1)=Ax(k)+Bu(k)+B_{d}d(k)+B_{f}f(k)\\ y(k)=Cx(k)+Du(k)+D_{d}d(k)+D_{f}f(k) \end{array}\right.$$

where $x(k)\in {\mathbb{R}}^{n}$, $u(k)\in {\mathbb{R}}^{p}$, $y(k)\in {\mathbb{R}}^{q}$, $d(k)\in {\mathbb{R}}^{l}$, and $f(k)\in {\mathbb{R}}^{m}$ are vectors representing the state, measurement output, control input, unknown input (including disturbance and noise), and fault, respectively. d(k) is assumed to be bounded by the $l_{2}$-norm. The matrices A, B, $B_{d}$, $B_{cf}$, C, D, $D_{d}$, and $D_{f}$ are known and have approximate dimensions.

Generally, DoS attacks can occur in both the network from the control unit to the actuators and the one from the sensors to the control/FD unit. These attacks can be achieved through timeout or network status monitoring. To address DoS attacks from the controller to the actuators, various robust control schemes can be used, and significant progress has been made [20,21]. Thus, the main challenge lies in the FD problems for NCSs with DoS attacks in the sensor-to-controller/FD unit network.

Taking into account the DoS attacks, the output after network transmission can be defined as follows:

(2) $$z(k)=\left\{\begin{array}{ll} y(k-1), & \;DoS\;\mathrm{attack}\;\mathrm{occurs}\;\mathrm{at}\;k\\ y(k),\; & \;\mathrm{no}\;DoS\;\mathrm{attack}\;\mathrm{at}\;k \end{array}\right.$$

Combining (1) and (2), we can derive the following LTI process with DoS attacks:

(3) $$\left\{\begin{array}{ll} x(k+1)=Ax(k)+Bu(k)+B_{d}d(k)+B_{f}f(k) & \\ z(k)=Cx(k)+Du(k)+D_{d}d(k)+D_{f}f(k), & DoS\;\mathrm{attack}\;\mathrm{occurs}\;\mathrm{at}\;k\\ z(k)=z(k-1), & \mathrm{no}\;DoS\;\mathrm{attack}\;\mathrm{at}\;k \end{array}\right.$$

A typical FD approach consists of two stages: residual generation and residual evaluation. In order to generate the residual, both the input/output data and the discrete-time system model are necessary. For a given parity space order s, the following parity relation holds for systems without DoS attacks

(4) $$y_{s}(k)-H_{us}u_{s}(k)=H_{os}x(k-s)+H_{ds}d_{s}(k)+H_{fs}f_{s}(k)$$

where $\xi_{s}(k)={\left[\begin{array}{cccc} \xi^{T}(k-s) & \xi^{T}(k-s+1) & \ldots & \xi^{T}(k) \end{array}\right]}^{T},\;\xi =u,y,d,f$, $H_{os}=\left[\begin{array}{c} C\\ CA\\ \vdots \\ CA^{s} \end{array}\right]$, $H_{us}=\left[\begin{array}{ccccc} D & 0 & 0 & \ldots & 0\\ CB & D & 0 & \ldots & 0\\ CAB & CB & D & \ldots & 0\\ \vdots & \ddots & \ddots & \ddots & \vdots \\ CA^{s-1}B & \ldots & CAB & CB & D \end{array}\right]$.

The matrices $H_{ds}$ and $H_{fs}$ are constructed by replacing {B,D} with $\left\{B_{d},D_{d}\right\}$ and $\left\{B_{f},D_{f}\right\}$ in $H_{us}$, respectively.

In the case of measurement DoS attacks, the traditional parity space-based residual generator can be selected as

(5) $$r_{t}(k)=PN_{os}(z_{s}(k)-H_{us}u_{s}(k))$$

where $N_{os}\in {\mathbb{R}}^{\gamma \times (s+1)q}$ is the base matrix of all possible parity space matrices that satisfies $N_{os}H_{os}=0$.

Regardless of the DoS attack, an $H_{-}/H_{2}$ scheme is introduced for the optimal design of the parity space matrix P, as follows:

(6) $$J_{P}=\underset{P}{\max}\frac{{\left\|P{\bar{H}}_{fs}\right\|}_{-}}{{\left\|P{\bar{H}}_{ds}\right\|}_{2}}$$

where ${\bar{H}}_{fs}=N_{os}H_{fs},\;{\bar{H}}_{ds}=N_{os}H_{ds}$. ${\left\|P{\bar{H}}_{fs}\right\|}_{-}$ represents the minimum nonzero singular value of the matrix $P{\bar{H}}_{fs}$, and induced norm ${\left\|P{\bar{H}}_{ds}\right\|}_{2}$ is defined as

$${\left\|P{\bar{H}}_{ds}\right\|}_{2}=\underset{d\neq 0}{\sup}\frac{\left\|P{\bar{H}}_{ds}d_{s}(k)\right\|}{\left\|d_{s}(k)\right\|}.$$

Following the derivation in [27], an optimal solution for the optimization problem (6) can be determined using Lemma 1.

**Lemma 1**  ([27])**.** *Given the LTI system (1), the optimization problem (6) can be solved by setting* $P=S^{-1}U^{T}$*, resulting in* $J_{P}={\left\|S^{-1}U^{T}{\bar{H}}_{fs}\right\|}_{-}$.

Here, the matrices S and U are obtained using the singular value decomposition (SVD) technique, where ${\bar{H}}_{ds}=U\Sigma V^{T}$, $UU^{T}=I$, $V^{T}V=I$, and Σ=[S 0]. The diagonal matrix S is defined as $S=diag\{\sigma_{1},\sigma_{2},\ldots \}$.

After generating the residual, the most widely used residual evaluation function is adopted as

(7) $$J_{t}(k)=r_{t}^{T}(k)r_{t}(k)$$

The corresponding threshold for $J_{t}(k)$ can be determined by $J_{th}=\underset{f=0,d}{\sup}J_{t}(k)$. In this case, the FD results can be obtained by applying

(8) $$\left\{\begin{array}{l} J_{t}(k)\geq J_{th},\;\mathrm{fault}\;\mathrm{alarm}\\ J_{t}(k)<J_{th},\;\mathrm{no}\;\mathrm{fault} \end{array}\right.$$

However, the presence of DoS attacks can greatly affect the accuracy of the output $z_{s}(k)$, making it difficult to match the real output $y_{s}(k)$ at every time step. This means that the parity relation (4) may not always hold true by replacing $y_{s}(k)$ with $z_{s}(k)$, resulting the residual $r_{t}(k)$ that takes into account the DoS attacks in

(9) $$r_{t}(k)=PN_{os}\left[y_{s}(k)-H_{us}u_{s}(k)\right]-PN_{os}\left[y_{s}(k)-z_{s}(k)\right]$$

Substituting (4) into (9) yields

(10) $$r_{t}(k)=PN_{os}\left[H_{ds}d_{s}(k)+H_{fs}f_{s}(k)\right]-PN_{os}\left[y_{s}(k)-z_{s}(k)\right]$$

It is important to note that the residual $r_{t}(k)$ not only includes information about the unknown input $d_{s}(k)$ and fault $f_{s}(k)$, but also the transmission error $\left[y_{s}(k)-z_{s}(k)\right]$ caused by the DoS attacks. Therefore, using the residual evaluation scheme (8) without considering the transmission error caused by DoS attacks may lead to poor FD performance, resulting in a high false alarm rate (FAR) or missed detection rate (MDR). These observations have motivated us to propose a parity space-based FD scheme for NCSs that are vulnerable to DoS attacks, in order to achieve better detection performance.

**Remark 1.**  *The impact of a DoS attack on the communication link between the sensor and the FD unit is typically represented as either packet loss or packet repetition (2). This is a commonly used model in NCSs* [14,15,16]*, as it effectively captures the primary consequence of a successful DoS attack on data availability and timing. However, real DoS attacks can exhibit more complex patterns, such as replay attacks, adaptive attacks, and coordinated network-level attacks. Despite these variations, the main challenge posed by a DoS attack remains the same: the FD unit is unable to reliably receive the correct output at the expected time. The proposed method is designed to be resilient against different patterns of packet loss. Further research is needed to analyze its performance under other types of DoS attacks.*

## 3. Main Results

### 3.1. Parity Relations for NCSs Under DoS Attacks

Recall that the traditional parity relation (4) is satisfied only if $z_{s}(k)=y_{s}(k)$ when all the latest (s+1) outputs in $y_{s}(k)$ are available for FD unit, which is no longer applicable for DoS attack scenarios. The main task is to establish a parity relation with DoS attacks into account.

Denote the latest (s+1) available outputs for FD unit as

(11) $$z_{d,s}(k_{i})={\left[\begin{array}{cccc} z^{T}(k_{i-s}) & z^{T}(k_{i-s+1}) & \ldots & z^{T}(k_{i}) \end{array}\right]}^{T}$$

where $k_{i-s},\;k_{i-s+1},\;\ldots ,\;k_{i}$ representing the instants corresponding to each component of the output through the communication network without DoS attacks.

Despite the different robust control scheme, the control signal u(k) during the time window $k_{i}$ can be assumed to be available for the FD unit through the network from sensors to the FD unit when the instant is $k_{i}$. Let $s_{k_{i}}=k_{i}-k_{i-s}$, and define

(12) $$u_{s_{k_{i}}}(k_{i})={\left[\begin{array}{cccc} u^{T}(k_{i-s}) & u^{T}(k_{i-s}+1) & \ldots & u^{T}(k_{i}) \end{array}\right]}^{T}$$

The vectors $d_{s_{k_{i}}}(k_{i})$ and $f_{s_{k_{i}}}(k_{i})$ can be obtained by replacing u(⋅) with d(⋅) and f(⋅) in $u_{s_{k_{i}}}(k_{i})$, respectively.

However, due to the presence of DoS attacks in the output-to-FD unit, the outputs y(k−1) and y(k) for adjacent instants k−1 and k may not always be available. To account for this, we consider two latest available outputs $z(k_{i-1})$ and $z(k_{i})$ for FD unit, with corresponding instants denoted by $k_{i-1}$ and $k_{i}$, where $k_{i}-k_{i-1}\geq 1$. It is easy to obtain

(13) $$z(k_{i-1})=Cx(k_{i-1})+Du(k_{i-1})+D_{d}d(k_{i-1})+D_{f}f(k_{i-1})$$

(14) $$\begin{array}{ll} z(k_{i})= & CA^{k_{i}-k_{i-1}}x(k_{i-1})+\sum_{j=0}^{k_{i}-k_{i-1}-1}CA^{k_{i}-k_{i-1}-j-1}Bu(k_{i-1}+j)+Du(k_{i})\\ & \;+\sum_{j=0}^{k_{i}-k_{i-1}-1}CA^{k_{i}-k_{i-1}-j-1}B_{d}d(k_{i-1}+j)+D_{d}d(k_{i})\\ & \;+\sum_{j=0}^{k_{i}-k_{i-1}-1}CA^{k_{i}-k_{i-1}-j-1}B_{f}f(k_{i-1}+j)+D_{f}f(k_{i}) \end{array}$$

Expanding the results (13) and (14) to include the instants $k_{i-s}$ to $k_{i}$, we obtain the following parity relation for NCSs under DoS attacks

(15) $$z_{d,s}(k_{i})-H_{us}^{i}u_{s_{k_{i}}}(k_{i})=H_{os}^{i}x(k_{i-s})+H_{ds}^{i}d_{s_{k_{i}}}(k_{i})+H_{fs}^{i}f_{s_{k_{i}}}(k_{i})$$

where $H_{os}^{i}={\left[\begin{array}{ccccc} C^{T} & {\left(CA^{\eta_{is}^{1}}\right)}^{T} & \ldots & {\left(CA^{\eta_{is}^{s-1}}\right)}^{T} & {\left(CA^{\eta_{is}^{s}}\right)}^{T} \end{array}\right]}^{T}$, $\eta_{is}^{j}=k_{i-s+j}-k_{i-s}$, 2≤j<s. $H_{us}^{i}={\left[\begin{array}{cccc} {\left(H_{us}^{i}\right)}_{1}^{T} & {\left(H_{us}^{i}\right)}_{2}^{T} & \ldots & {\left(H_{us}^{i}\right)}_{s}^{T} \end{array}\right]}^{T}$, ${\left(H_{us}^{i}\right)}_{j}$ represents the jth block matrices for $H_{us}^{i}$, with ${\left(H_{us}^{i}\right)}_{1}=\left[\begin{array}{cccc} D & 0 & \ldots & 0 \end{array}\right]$, ${\left(H_{us}^{i}\right)}_{2}=\left[\begin{array}{ccccccccc} CA^{\eta_{is}^{1}-1}B & CA^{\eta_{is}^{1}-2}B & \ldots & CAB & CB & D & 0 & \ldots & 0 \end{array}\right]$, ${\left(H_{us}^{i}\right)}_{j}=\left[\begin{array}{ccccccccc} CA^{\eta_{is}^{j-1}-1}B & CA^{\eta_{is}^{j-1}-2}B & \ldots & CAB & CB & D & 0 & \ldots & 0 \end{array}\right]$, 2≤j<s, ${\left(H_{us}^{i}\right)}_{s+1}=\left[\begin{array}{cccccc} CA^{\eta_{is}^{s}-1}B & CA^{\eta_{is}^{s}-2}B & \ldots & CAB & CB & D \end{array}\right]$. The matrices $H_{ds}^{i}$ and $H_{fs}^{i}$ are constructed by replacing (B,D) with $\left(B_{d},D_{d}\right)$ and $\left(B_{f},D_{f}\right)$ in $H_{us}^{i}$, respectively.

The parity relation (15) is established between the state $x(k_{i-s})$, the most recent available (s+1) measurement $z_{d,s}(k_{i})$, the input $u_{s_{k_{i}}}(k_{i})$, the unknown input $d_{si}(k)$, and the fault $f_{si}(k)$ during the time window of length $\left(k_{i}-k_{i-s}\right)$ considering the DoS attacks. In comparison to the traditional relation (4), the proposed parity relation (15) utilizes all available input/output information from instant $k_{i-s}$ to instant $k_{i}$ without introducing any transmission errors caused by DoS attacks. While the traditional method in [26] introduces such errors through data substitution, the proposed method has a theoretical advantage over the traditional method because it eliminates transmission errors caused by DoS attacks.

### 3.2. Residual Generator

To eliminate residual errors caused by DoS attacks, we propose the following residual generator

(16) $$r(k_{i})=P_{k_{i}}N_{os}^{i}(z_{d,s}(k_{i})-H_{us}^{i}u_{s_{k_{i}}}(k_{i}))$$

where the matrix $N_{os}^{i}\in {\mathbb{R}}^{\gamma_{i}\times (1+s)q}$ is the basis matrix of all the $\gamma_{i}\times (1+s)q$ matrices that satisfy $N_{os}^{i}H_{os}^{i}=0$. The parity space matrix $P_{k_{i}}$ will also need to be designed.

By substituting the result $N_{os}^{i}H_{os}^{i}=0$ and the parity relation (15) into (16), we can obtain the following residual

(17) $$r(k_{i})=P_{k_{i}}N_{os}^{i}(H_{ds}^{i}d_{s_{k_{i}}}(k_{i})+H_{fs}^{i}f_{s_{k_{i}}}(k_{i}))$$

Despite the DoS attacks, the proposed residual (17) contains almost all of the necessary information regarding faults and unknown inputs during the time window from $k_{i-s}$ to $k_{i}$. Additionally, unlike the residual used in [26], the proposed residual is not affected by transmission errors caused by DoS attacks, such as $\left[y_{s}(k)-z_{s}(k)\right]$.

To further clarify, define ${\bar{H}}_{ds}^{i}=N_{os}^{i}H_{ds}^{i}$ and ${\bar{H}}_{fs}^{i}=N_{os}^{i}H_{fs}^{i}$. This allows us to express the residual as follows:

(18) $$r(k_{i})=P_{k_{i}}({\bar{H}}_{ds}^{i}d_{s_{k_{i}}}(k_{i})+{\bar{H}}_{fs}^{i}f_{s_{k_{i}}}(k_{i}))$$

The next task is to design the parity space matrix $P_{k_{i}}$ in (18). Let ${\left\|P_{k_{i}}{\bar{H}}_{fs}^{i}\right\|}_{-}$ represent the minimum nonzero singular value of $P_{k_{i}}{\bar{H}}_{fs}^{i}$. Additionally, the following induced norms are introduced to quantitatively evaluate the robustness of the residual against the unknown input:

$${\left\|P_{k_{i}}{\bar{H}}_{ds}^{i}\right\|}_{2}=\underset{d_{s_{k_{i}}}\neq 0}{\sup}\frac{\left\|P_{k_{i}}{\bar{H}}_{ds}^{i}d_{s_{k_{i}}}(k_{i})\right\|}{\left\|d_{s_{k_{i}}}(k_{i})\right\|}$$

As a result, the following $H_{-}/H_{2}$ scheme is presented for the optimal design of the parity space matrix $P_{k_{i}}$:

(19) $$J_{P_{k_{i}}}=\underset{P_{k_{i}}}{\max}\frac{{\left\|P_{k_{i}}{\bar{H}}_{fs}^{i}\right\|}_{-}}{{\left\|P_{k_{i}}{\bar{H}}_{ds}^{i}\right\|}_{2}}$$

Extending Lemma 1 to this optimization problem (19), we obtain the following Theorem 1:

**Theorem 1.**  *For a given NCS (3) subject to DoS attacks, $P_{k_{i}}=S_{k_{i}}^{-1}U_{k_{i}}^{T}$ solves the optimization problem (19) and result $J_{P_{k_{i}}}$ in $J_{P_{k_{i}}}={\left\|S_{k_{i}}^{-1}U_{k_{i}}^{T}{\bar{H}}_{fs}^{i}\right\|}_{-}$.*

The matrices $S_{k_{i}}$ and $U_{k_{i}}$ are obtained by performing an SVD on ${\bar{H}}_{ds}^{i}$, which can be written as ${\bar{H}}_{ds}^{i}=U_{k_{i}}\Sigma_{k_{i}}V_{k_{i}}^{T}$, where $U_{k_{i}}U_{k_{i}}^{T}=I$, $V_{k_{i}}^{T}V_{k_{i}}=I$, and $\Sigma_{k_{i}}=\left[\begin{array}{cc} S_{k_{i}} & 0 \end{array}\right]$, with $S_{k_{i}}=\mathrm{diag}(\sigma_{k_{i}1},\sigma_{k_{i}2},\ldots ,\sigma_{k_{i}\gamma_{i}})$, where $\sigma_{k_{i}1},\sigma_{k_{i}2},\ldots ,\sigma_{k_{i}\gamma_{i}}$ are nonzero singular values.

**Proof.**  Let $P_{k_{i}}=X_{k_{i}}S_{k_{i}}^{-1}U_{k_{i}}^{T}$. Substituting this into (19) yields

$$\frac{{\left\|P_{k_{i}}{\bar{H}}_{fs}^{i}\right\|}_{-}}{{\left\|P_{k_{i}}{\bar{H}}_{ds}^{i}\right\|}_{2}}=\frac{{\left\|X_{k_{i}}S_{k_{i}}^{-1}U_{k_{i}}^{T}{\bar{H}}_{fs}^{i}\right\|}_{-}}{{\left\|X_{k_{i}}S_{k_{i}}^{-1}U_{k_{i}}^{T}{\bar{H}}_{ds}^{i}\right\|}_{2}}=\frac{{\left\|X_{k_{i}}S_{k_{i}}^{-1}U_{k_{i}}^{T}{\bar{H}}_{fs}^{i}\right\|}_{-}}{{\left\|X_{k_{i}}\right\|}_{2}}.$$

Due to ${\left\|X_{k_{i}}\right\|}_{+}={\left\|X_{k_{i}}\right\|}_{2}$ and

$$\frac{{\left\|X_{k_{i}}S_{k_{i}}^{-1}U_{k_{i}}^{T}{\bar{H}}_{fs}^{i}\right\|}_{-}}{{\left\|X_{k_{i}}\right\|}_{2}}\leq \frac{{\left\|X_{k_{i}}\right\|}_{+}{\left\|S_{k_{i}}^{-1}U_{k_{i}}^{T}{\bar{H}}_{fs}^{i}\right\|}_{-}}{{\left\|X_{k_{i}}\right\|}_{2}},$$

we further obtain $\frac{{\left\|P_{k_{i}}{\bar{H}}_{fs}^{i}\right\|}_{-}}{{\left\|P_{k_{i}}{\bar{H}}_{ds}^{i}\right\|}_{2}}\leq {\left\|S_{k_{i}}^{-1}U_{k_{i}}^{T}{\bar{H}}_{fs}^{i}\right\|}_{-}$.

Let $X_{i}=I$, and it is easy to get $\frac{{\left\|P_{k_{i}}{\bar{H}}_{fs}^{i}\right\|}_{-}}{{\left\|P_{k_{i}}{\bar{H}}_{ds}^{i}\right\|}_{2}}={\left\|S_{k_{i}}^{-1}U_{k_{i}}^{T}{\bar{H}}_{fs}^{i}\right\|}_{-}$, which implies $J_{P_{k_{i}}}={\left\|S_{k_{i}}^{-1}U_{k_{i}}^{T}{\bar{H}}_{fs}^{i}\right\|}_{-}$. □

The result proves that $P_{k_{i}}=S_{k_{i}}^{-1}U_{k_{i}}^{T}$ solves the optimization problem (19). If there is singular values approximately zero, the matrix can be set $S_{k_{i}}=\mathrm{diag}(\sigma_{k_{i}1},\sigma_{k_{i}2},\ldots ,\sigma_{k_{i}\tau_{i}})$$\tau_{i}\leq \gamma_{i}$, resulting in $P_{k_{i}}\in {\mathbb{R}}^{\tau_{i}\times \gamma_{i}}$.

After applying Theorem 1 to determine the matrix $P_{k_{i}}$, the online residual $r(k_{i})$ can be updated using (16). The proposed residual takes into account all available inputs $u_{s_{k_{i}}}(k_{i})$ from instant $k_{i-s}$ to instant $k_{i}$, as well as the latest (s+1) available measurement outputs $z_{d,s}(k_{i})$ for the FD unit. Furthermore, this residual is characterized by its ability to decouple from transmission errors caused by DoS attacks.

If the frequency of DoS attacks is low, the real-time residual $r(k_{i})$ will remain the same as the traditional parity space-based residual $r_{e}(k)$ for most instances. In this case, the residual $r(k_{i})$ will only be updated when a DoS attack occurs. To achieve this, we define

(20) $$g(k)=\left\{\begin{array}{l} 0,\;\mathrm{no}\;\mathrm{DoS}\;\mathrm{attack}\;\mathrm{occurs}\;\mathrm{for}\;k-j,\;\forall j=0,1,\ldots ,s\\ 1,\;\mathrm{DoS}\;\mathrm{attack}\;\mathrm{occurs}\;\mathrm{for}\;k\\ 2,\;\mathrm{otherwise} \end{array}\right.$$

The value g(k)=0 indicates that no DoS occurred during the instant (k−s) to k, while g(k)=1 represents a dropout of the output at the current instant k. If g(k)=2, it means that the current output y(k) is available, but at least one previous output y(k−i), where i=1,2,…,s, is unavailable.

Using this information, the residual r(k) for the current instant k can be reconstructed as follows

(21) $$r(k)=\left\{\begin{array}{ll} r_{t}(k), & g(k)=0\\ r(k-1), & g(k)=1\\ r(k_{i}), & g(k)=2 \end{array}\right.$$

The residual r(k) remains decoupled from transmission errors caused by DoS attacks, while for the DoS attack-free case, it is replaced with the traditional parity space-based residual with time invariant parameter matrices.

**Remark 2.**  *It should be noted that the value of s in (15) represents the number of the most recent available measurements for the FD unit, rather than the length of the time window for input/output data in the traditional parity space approach. The actual length of the time window for input/output data is $\left(k_{i}-k_{i-s}\right)$, which is determined by the number of DoS attacks that occur in the communication network between the output and FD unit at the instants $k_{i-s}$ and $k_{i}$. In the case where $k_{i}-k_{i-1}=1,\;\forall \;i=1,2,\ldots$, the residual will revert to the traditional residual (5).*

### 3.3. Residual Evaluation with Unified Threshold

Under the DoS attacks, the residual r(k) in (21) has a time-varying dimension because the window length $\left(k_{i}-k_{i-s}\right)$ changes, resulting in a time-varying matrix $P_{k_{i}}$. To construct a decision logic that is comparable across different window lengths, we define the following evaluation function for DoS attack scenarios:

(22) $$J(k_{i})=\frac{s+1}{k_{i}-k_{i-s}+1}\left[r^{T}(k_{i})r(k_{i})\right]$$

The parameter $\frac{s+1}{k_{i}-k_{i-s}+1}$ adjusts for the varying length of $r(k_{i})$ for the DoS attacks, ensuring that the expected magnitude of $J(k_{i})$ under fault-free conditions does not depend on $\left(k_{i}-k_{i-s}\right)$.

In the absence of a DoS attack, the value of $k_{i}-k_{i-s}+1$ is equal to s+1, resulting in the following evaluation function:

(23) $$J_{t}(k)=r_{t}^{T}(k)r_{t}(k)$$

Thus, we get the following the evaluation function:

(24) $$J(k)=\left\{\begin{array}{ll} J_{t}(k), & g(k)=0\\ J(k-1), & g(k)=1\\ J(k_{i}), & g(k)=2 \end{array}\right.$$

The evaluation function J(k) is derived from the fact that the magnitude of $r(k_{i})$ increases linearly with the number of entries in $r(k_{i})$ when the underlying signals are bounded. As a result, the threshold can be chosen as a constant value that is independent of the attack pattern. The unknown input is assumed to be bounded by the $l_{2}$ norm, defined as $||d(k)|{|}^{2}<\delta_{d}$. This assumption is commonly used in parity space-based FD methods [27] and does not rely on a probabilistic distribution of the unknown input.

In the fault-free case without DoS attacks, we obtain

$$\left\|r_{t}(k)\right\|\leq \left\|P{\bar{H}}_{ds}\right\|\cdot \left\|d_{s}(k)\right\|$$

Then,

$$\begin{array}{ll} J_{t}(k)=r_{t}^{T}(k)r_{t}(k) & \leq {\left\|d_{s}(k)\right\|}^{2}\cdot {\left\|P{\bar{H}}_{ds}\right\|}^{2}\\ & \leq (s+1)\delta_{d}\cdot {\left\|P{\bar{H}}_{ds}\right\|}^{2} \end{array}$$

Additionally, in the fault-free case under the DoS attack, it is easy to obtain

$$\left\|r(k_{i})\right\|\leq \left\|P_{k_{i}}{\bar{H}}_{ds}^{i}\right\|\cdot \left\|d_{s_{k_{i}}}(k_{i})\right\|$$

Similarly,

$$J(k_{i})=\frac{s+1}{k_{i}-k_{i-s}+1}\left[r^{T}(k_{i})r(k_{i})\right]\leq \frac{s+1}{k_{i}-k_{i-s}+1}{\left\|d_{s_{k_{i}}}(k_{i})\right\|}^{2}\cdot {\left\|P_{k_{i}}{\bar{H}}_{ds}^{i}\right\|}^{2}$$

$$\leq (s+1)\delta_{d}\cdot {\left\|P_{k_{i}}{\bar{H}}_{ds}^{i}\right\|}^{2}$$

If we select the matrix $P_{k_{i}}$ to satisfy $\sup{\left\|P{\bar{H}}_{ds}\right\|}^{2}=\underset{k_{i}}{\sup}{\left\|P_{k_{i}}{\bar{H}}_{ds}^{i}\right\|}^{2}=\delta_{p}$, it is reasonable to set the threshold as $J_{th}=(s+1)\delta_{d}\delta_{p}$. Alternatively, when $\sup{\left\|P{\bar{H}}_{ds}\right\|}^{2}\leq \delta_{p1}$ and $\underset{k_{i}}{\sup}{\left\|P_{k_{i}}{\bar{H}}_{ds}^{i}\right\|}^{2}\leq \delta_{p2}$, we can select the threshold as $J_{th}=(s+1)\delta_{d}\cdot \max\left\{\delta_{p1},\delta_{p2}\right\}$. The threshold $J_{th}$ can be precomputed offline by utilizing the known bounds of the unknown input and the optimized parity matrix $P_{k_{i}}{\bar{H}}_{ds}^{i}$.

### 3.4. Fault Detectability Analysis

It is easy to get the necessary condition for detection of fault f(k) as follows

(25) $$\left\|P_{k_{i}}{\bar{H}}_{fs}^{i}f_{s_{k_{i}}}(k_{i})\right\|\neq 0$$

The threshold $J_{th}$ applies to all possible evaluation functions, and in the absence of any faults, the FAR is theoretically zero. To ensure that a fault f(k) can be detected, its contribution to the evaluation function J(k) must exceed the threshold $J_{th}$ in the worst-case scenario. A sufficient condition for detecting the fault f(k) is

(26) $$\left\|P_{k_{i}}{\bar{H}}_{fs}^{i}f_{s_{k_{i}}}(k_{i})\right\|\geq 2\sqrt{J_{th}}$$

Conditions (25) and (26) quantify the necessary and sufficient conditions for detecting a fault f(k).

### 3.5. Real-Time FD Algorithms

To reduce the burden of online computation, the commonly used matrices $H_{os}^{i}$, $H_{ds}^{i}$, $H_{fs}^{i}$, and $P_{k_{i}}$ can be pre-computed and stored in the FD unit based on the frequency and characteristics of DoS attacks.

When the online detection performance cannot be guaranteed, a smoothing function can be introduced to improve it. For instance, over the N available functions J(k), we suggest using the following residual evaluation function:

(27) $$J_{a}(k)=\frac{1}{N+1}\sum_{j=0}^{N}J(k_{i-j})$$

It is reasonable to choose the threshold for $J_{a}(k_{i})$ as

(28) $$J_{th,a}:=\underset{f=0,k_{i}}{\sup}\left\{J_{a}(k_{i})\right\}$$

As a result, the following FD logic can be given as

(29) $$\left\{\begin{array}{l} J_{a}(k)\geq J_{th,a},\;\mathrm{fault}\;\mathrm{alarm}\\ J_{a}(k)<J_{th,a},\;\mathrm{no}\;\mathrm{fault} \end{array}\right.$$

Based on the residual r(k), the evaluation function J(k) and $J_{a}(k)$ can be updated using (24) and (27), respectively. The final FD result can be obtained by applying the FD logic (29). The proposed parity space-based FD scheme for NCSs with DoS attacks is summarized in Algorithm 1.
**Algorithm 1.** The parity space-based FD scheme for NCSs with DoS attacksStep 1. Set the parity space order s$\;\mathrm{and}\;\mathrm{the}\;\mathrm{initial}\;\mathrm{instant}\;k_{0}\geq s$ for FD.Step 2. Update $z_{d,s}(k_{i})$$\;\mathrm{and}\;u_{s_{k_{i}}}(k_{i})$, and compute or find the pre-computed matrices $N_{os}^{i}$$,\;H_{os}^{i}$$,\;H_{us}^{i}$$,\;H_{ds}^{i}$$,\;H_{fs}^{i}$$,\;{\bar{H}}_{ds}^{i}$$,\;\mathrm{and}\;{\bar{H}}_{fs}^{i}$.$\mathrm{Step}\;3.\;\mathrm{Perform}\;\mathrm{SVD}\;\mathrm{on}\;{\bar{H}}_{ds}^{i}$$\;\mathrm{to}\;\mathrm{obtain}\;U_{k_{i}}$$\;\mathrm{and}\;S_{k_{i}}$.$\mathrm{Step}\;4.\;\mathrm{Compute}\;\mathrm{parity}\;\mathrm{space}\;\mathrm{matrix}\;P_{k_{i}}$ using Theorem 1.Step 5. Update r(k), J(k)$\;\mathrm{and}\;J_{a}(k)$ using (21), (24), and (27). Step 6. Use the FD logic (29) to obtain the FD result.

### 3.6. Computational Complexity Analysis

One practical concern is the computational burden introduced by SVD and adaptive windowing. If the frequency and duration of DoS attacks are fixed and available, the matrices $P_{k_{i}}N_{os}^{i}$ and $H_{us}^{i}$ can be pre-calculated and stored in the FD unit for real-time calculation and direct query.

In this case, the main online computational burden lies in updating r(k), J(k), and $J_{a}(k)$ (if necessary). The main computational complexity is $O_{1}=O\left((s+1)\left[(k_{i}-k_{i-s}+1)pq+\tau_{i}q+q\right]+\tau_{i}+N+1\right)$.

However, if the matrices $P_{k_{i}}$, $N_{os}^{i}$, and $H_{us}^{i}$ cannot be stored offline, there will be additional computation required. This includes updating the parity space matrices $H_{os}^{i}$, $H_{ds}^{i}$, $H_{us}^{i}$, $N_{os}^{i}$, ${\bar{H}}_{d,s}$, and $P_{k_{i}}N_{os}^{i}$. Since $CA^{i}(1\leq i\leq k_{i}-k_{i-s})$ does not require repeated calculations and there is structural similarity between the matrices $H_{os}^{i}$, $H_{us}^{i}$, and $H_{us}^{i}$, the computational complexity of these three matrices is approximately $O_{2}=O((k_{i}-k_{i-s})qn^{2}+(k_{i}-k_{i-s}-1)(qnp+qnl))$.

The complexity of the null matrix $N_{os}^{i}$ is approximately $O_{3}=O\left(max\left\{\gamma_{i}^{3},{\left[(s+1)l\right]}^{3}\right\}\right)=O\left({\left(s+1\right)}^{3}l^{3}\right)$ based on SVD, or $O_{3}=O\left(2\gamma_{i}{\left[(s+1)l\right]}^{2}-\frac{2}{3}{\left[(s+1)l\right]}^{3}\right)$ based on QR decomposition. The complexity of ${\bar{H}}_{ds}^{i}=N_{os}^{i}H_{ds}^{i}$ is approximately $O_{4}=O\left(\gamma_{i}(k_{i}-k_{i-s}+1)q(s+1)l\right)$.

The parity space matrix $P_{k_{i}}$ is obtained via Theorem 1, which requires an SVD on the matrix ${\bar{H}}_{d,s}\in {\mathbb{R}}^{\gamma_{i}\times (s+1)l}$. The SVD complexity is approximately $O_{5}=O\left(max\left\{\gamma_{i}^{3},{\left[(s+1)l\right]}^{3}\right\}\right)=O\left({\left(s+1\right)}^{3}l^{3}\right)$. The complexity of $P_{k_{i}}=S_{k_{i}}^{-1}U_{k_{i}}^{T}$ and $P_{k_{i}}N_{os}^{i}$ is approximately $O_{6}=O\left(\tau_{i}\right)+O\left(\tau_{i}^{2}\gamma_{i}\right)$ and $O_{7}=O\left(\tau_{i}\gamma_{i}(k_{i}-k_{i-s}+1)q\right)$.

In the case where updating the matrices $P_{k_{i}}$, $N_{os}^{i}$, and $H_{us}^{i}$ is necessary, the overall computational complexity is approximately $O_{all}=O_{1}+O_{2}+O_{3}+O_{4}+O_{5}+O_{6}+O_{7}$.

The adaptive window length s can be adjusted to improve the performance of the FD system. If the system dimension increases, choosing a smaller parity order s can sacrifice some performance for faster processing. This is possible because the adaptive relation proposed in this study is effective for any s≥0.

## 4. Simulation Results and Analysis

To validate the effectiveness of the proposed FD approach, we use Matlab 2021b to simulate a linearized longitudinal model of an AUV, specifically the Subzero II ROV [28,29]. Set

$$x(k)={\left[\begin{array}{ccccccc} \Delta v_{u} & \Delta v_{w} & \Delta q & \Delta \theta & \Delta Z & \Delta n_{r} & \Delta \delta_{s} \end{array}\right]}^{T},y(k)={\left[\begin{array}{cc} \Delta v_{u} & \Delta Z \end{array}\right]}^{T},\;u(k)={\left[\begin{array}{cc} \Delta M_{d} & \Delta \delta_{sd} \end{array}\right]}^{T},d(k)={\left[\begin{array}{cc} d_{1} & d_{2} \end{array}\right]}^{T},f(k)={\left[\begin{array}{ccc} f_{1} & f_{2} & f_{3} \end{array}\right]}^{T}$$

where Δ(⋅) represents the deviation of the variable (⋅), with $v_{u},\;v_{w},\;q,\;\theta ,\;Z,\;n_{r},\;\delta_{s}$ denoting the forward speed, vertical speed, pitch rate, pitch angle, depth, propeller rotation speed, and control surface deflection, respectively. The variables $\Delta M_{d}$ and $\Delta \delta_{sd}$ represent the motor command and control surface command, while $d_{1}$ and $d_{2}$ denote disturbances and noise. The outputs $\Delta v_{u}$ and ΔZ are measured by sonar and depthometer, respectively, and the faults $f_{1},\;f_{2},\;f_{3}$ represent the actuator fault of the control surface, sonar fault, and depthometer fault.

The sampling period is set to T=1 s, and the parameters of the system model are chosen based on the values used in [28]. The resulting LTI system is as follows:

$$\left\{\begin{array}{l} x(k+1)=Ax(k)+Bu(k)+B_{d}d(k)+B_{f}f(k)\\ y(k)=Cx(k)+D_{d}d(k)+D_{f}f(k) \end{array}\right.$$

where

$$\begin{array}{l} A=I+T\left[\begin{array}{ccccccc} 0.14 & 0.82 & -0.1 & -0.21 & -1.7 & 0.0011 & -0.003\\ 0.18 & -20.0 & 2.6 & 4.1 & 48.0 & 0.00013 & 0.067\\ 0.94 & -110.0 & 14.0 & 21.0 & 240.0 & 0.00065 & 0.48\\ 0.087 & -10.0 & 1.4 & 2.9 & 23.0 & 0.000044 & 0.00044\\ 0.008 & -0.78 & 0.1 & -0.073 & 3.2 & 0.0000078 & 0.0017\\ -120.0 & 34.0 & -5.3 & -14.0 & -49.0 & -0.57 & -0.022\\ -0.97 & 110.0 & -14.0 & -22.0 & -260.0\; & -0.00067 & -0.74 \end{array}\right], \end{array}$$

$$B=T\left[\begin{array}{cc} 0.00035 & 0.0043\\ -0.00000013 & -0.15\\ -0.000014 & -0.76\\ -0.0000012 & -0.071\\ 0.000000067 & -0.0068\\ 0.055 & 0.0023\\ 0 & 0.81 \end{array}\right],C=\left[\begin{array}{ccccccc} 1 & 0 & 0 & 0 & 0 & 0 & 0\\ 0 & 0 & 0 & 0 & 1 & 0 & 0 \end{array}\right],B_{d}=B,$$

$$B_{f}=\left[\begin{array}{cc} B_{f1} & 0 \end{array}\right],\;D_{d}=I,\;D_{f}=\left[\begin{array}{cc} 0 & I \end{array}\right],\;B_{f1}=T{\left[\begin{array}{ccccccc} 0.0043 & -0.15 & -0.76 & -0.071 & -0.0068 & 0.0023 & 0.81 \end{array}\right]}^{T}.$$

A state observer-based feedback control law, $u(k)=K\hat{x}(k)$, is applied to ensure system stability with

$$K=\left[\begin{array}{ccccccc} -594.17 & 244.12 & -52.20 & -202.44 & 2088.00 & -1.96 & -1.77\\ 5.34 & 40.30 & -6.61 & -54.08 & 437.38 & 0.018 & 0.062 \end{array}\right]$$

Set the parity space order s=5, and the initial state

$$x(0)={\left[\begin{array}{ccccccc} 0.0013 & -0.0321 & -0.2147 & -0.0079 & -0.0027 & -0.0117 & 0.2650 \end{array}\right]}^{T}$$

To assess the effectiveness of the proposed FD method, we will use the following metrics for measuring detection performance.

Fault Detection Rate (FDR)/Recall:

$$FDR=\frac{TP}{TP+FN}$$

where TP represents the number of time steps in which faults are correctly detected, and FN represents the number of time steps in which fault alarms are missed.

Miss Detection Rate (MDR):

MDR=1−FDR

False Alarm Rate (FAR):

$$FAR=\frac{FP}{FP+TN}$$

where FP and TN represent the number of time steps with false alarms and the one without false alarms in the fault-free case, respectively.

Precision:

$$Precision=\frac{TP}{TP+FP}$$

F1-score:

$$F1=2\cdot \frac{\;\mathrm{Precision}\;\cdot \;\mathrm{Recall}\;}{\;\mathrm{Precision}\;+\;\mathrm{Recall}\;}$$

Detection Delay: This metric measures the average number of sampling steps between the occurrence of a fault and the first time the evaluation function exceeds the threshold, but only for successfully detected faults.

In this simulation, we compare our proposed scheme to the traditional parity space approach [26] and a Kalman filter (KF)-based scheme [27]. The KF scheme is a representative of observer/filter-based approaches.

The initial FD time is set to $k_{0}=20$. The simulation time is t=1000s. Additionally, it is assumed that the disturbance/noise, $d_{1}$ and $d_{2}$, are band-limited white noises with covariance matrices of $\Sigma_{d}=0.01I$. The following three faulty cases are considered under DoS attacks at different frequencies and last time steps.

Case 1: An actuator fault occurs in the control surface with DoS attacks at output instants k=240, 490, 740, 990, lasting for three time steps. The fault $f_{1}(k)$ is simulated as

$$f_{1}(k)=\left\{\begin{array}{ll} 0.5, & 200\leq k\leq 250\\ -0.5, & 500\leq k\leq 5500\\ 0, & otherwise \end{array}\right.$$

Case 2: A sensor fault occurs in sonar with the DoS attacks at output instants k=300, 800, lasting for two time steps. The fault $f_{2}(k)$ is simulated as

$$f_{2}(k)=\left\{\begin{array}{ll} 0.0005, & 200\leq k\leq 250\\ -0.0005, & 500\leq k\leq 5500,\\ 0, & otherwise \end{array}\right.$$

Case 3: A sensor fault occurs in depthometer with the DoS attacks at output instants k=300, 600, 900, lasting for four time steps. The fault $f_{3}(k)$ is simulated as

$$f_{3}(k)=\left\{\begin{array}{ll} 0.001, & 200\leq k\leq 250\\ -0.001, & 500\leq k\leq 5500,\\ 0, & otherwise \end{array}\right.$$

By applying Algorithm 1, the evaluation functions J(k) of the proposed FD method can be updated, and the threshold is computed as $J_{th}=0.225$. The evaluation function J(k) is denoted as $J_{1}(k)$ for case 1, $J_{2}(k)$ for case 2, and $J_{3}(k)$ for case 3. Additionally, we conduct the traditional parity space method [26]. Denote the residual evaluation function $J_{t}(k)$ as $J_{t1}(k)$ for case 1, $J_{t2}(k)$ for case 2, and $J_{t3}(k)$ for case 3. The threshold is also $J_{th}=0.225$. It should be noted that neither the proposed method nor the traditional parity space method utilizes a smoothing function.

In the KF-based scheme, the KF is designed for the nominal system using the known system matrices and covariances, with the goal of matching the norm bound of the unknown input d(k). The residual and evaluation function are then defined as follows:

$$r_{kf}(k)=y(k)-C\hat{x}(\left.k\right|k-1),r_{kf}(k)=\left[\begin{array}{c} r_{kf1}(k)\\ r_{kf2}(k) \end{array}\right],\;J_{kfj}(k)=\sum_{i=0}^{N_{kf}-1}\left[r_{kfj}^{T}(k-i)r_{kfj}(k-i)\right]$$

where j=1,2, $N_{kf}=10$. $J_{kfji}(k)$ represent the $J_{kfj}(k)$ in Case i, i=1,2,3. The thresholds for $J_{kf1}(k)$ and $J_{kf2}(k)$ are set at $J_{kf1,th}=0.047$ and $J_{kf2,th}=0.0282$, respectively, to achieve a 1% FAR in a fault-free scenario without any DoS attacks. If either the evaluation function $J_{kf1}(k)$ or $J_{kf2}(k)$ exceeds the threshold, a fault alarm is triggered. In the event of a DoS attack, the KF will be implemented using the last received output when current data is unavailable.

Table 1 presents a systematic comparison of the three methods. The proposed method stands out by effectively eliminating transmission errors through the reconstruction of the parity relation using an adaptive window. In contrast, both the traditional parity space method and the KF-based method rely on zero-order hold (ZOH) substitution, which introduces errors that cannot be differentiated from actual faults. This fundamental distinction is the reason why the proposed method achieves a unified threshold and a theoretically zero false alarm rate (FAR), while the other methods require the threshold to be adjusted based on the type of attack.

We tested all the three methods using the same types of attacks described in Cases 1–3. The FD results are shown in Figure 2, Figure 3, Figure 4, Figure 5, Figure 6, Figure 7, Figure 8, Figure 9 and Figure 10. Additionally, Table 2 presents the average metrics over the three fault cases (actuator fault, sonar fault, depthometer fault) under DoS attacks.

It is evident from Table 2 that both the proposed FD scheme and the traditional approach have high FDRs of 98.37%, while the KF-based FD scheme has a significantly lower FDR of 24.84%. These findings indicate that both parity space approaches outperform the KF-based method in terms of detection performance for a range of faulty scenarios, including both actuator and sensor faults.

Additionally, Table 2 demonstrates that the proposed scheme achieves a FAR of 0.91%, which is theoretically zero. This is because even though the fault does not occur at time step k, it remains present during $\left[k-s,\;k\right]$ or $\left[k-(k_{i}-k_{i-s}),\;k\right]$. In this scenario, the residual r(k) still contains information about the fault. For the time-varying parity space window without the fault information, the correct FD result will be obtained. In contrast, the traditional parity space scheme struggles to differentiate between faults and DoS attacks, regardless of the frequency and duration of the attack. These findings highlight the effectiveness of the proposed FD approach in avoiding false alarms caused by DoS attacks.

The traditional parity space method has a higher FAR of 3.64% compared to the proposed scheme. This is due to the introduction of transmission errors through the use of zero order hold substitution, which can be mistaken for faults. If the frequency of DoS attacks increases, the FAR will also increase accordingly. Both the proposed method and the traditional parity space method are able to detect faults immediately after they occur, with an average delay of approximately 0.5 sampling step. This is because the residual changes sharply once a fault occurs.

However, the KF-based method has a low FDR of 24.84%. It may not even trigger a fault alarm during the [200, 250] time interval in Case 3. This is partly because the lack of a $H_{-}/H_{\infty }$ performance index to improve the fault sensitivity of the residual. The FAR, which is pre-tuned to be 1% under no attacks, can increase to about 2.62% under DoS attacks due to the unreliability of the innovation sequence when outputs are missing.

The proposed method achieves a MDR of 1.63%, a FAR as low as 0.91%, and the highest F1 score of 95.41%. Simulation results demonstrate that the proposed residual, designed to detect faults in NCSs subject to DoS attacks, is able to decouple from transmission errors caused by DoS attacks in the sensor-to-FD unit. These results support the conclusion that the proposed parity space approach, with adaptive windowing and a unified threshold, has better FD performance than both the classical parity space method and a representative observer-based method (KF) for the simulated system under DoS attacks.

In this experiment, the matrices $P_{k_{i}}N_{os}^{i}$ and $H_{us}^{i}$ are pre-calculated and stored in the FD unit. The primary computational complexity is

$$\begin{array}{ll} O_{1} & =O\left((s+1)\left[(k_{i}-k_{i-s}+1)pq+\tau_{i}q+q\right]+\tau_{i}+N+1\right)\\ & =O\left(24(k_{i}-k_{i-s}+1)+12\tau_{i}+13+\tau_{i}\right) \end{array}$$

Considering the DoS attacks in Case 1, we have $k_{i}-k_{i-s}=8$, and compute the corresponding matrices with $\tau_{i}=4$, resulting in a computational complexity of $O_{1}=O\left(281\right)$.

Notably, the proposed method relies solely on input-output data and system matrices $\left(A,B,B_{d},B_{f},C,D,D_{d},D_{f}\right)$. The theoretical derivations in Section 3 are applicable to any LTI system with detectable faults, making the method suitable for LTI systems of any order subject to norm-bounded disturbance and noise. In the simulation example, an AUV is used to demonstrate the successful detection of faults under DoS attacks. This paper focuses on the theoretical derivation of the parity space scheme with time-varying windows for LTI systems, while the next step will be hardware-in-the-loop or real-plant tests. The current simulation results, combined with the theoretical guarantees, support the claim of broad applicability within the LTI systems.

The proposed parity space-based FD method is specifically designed to handle DoS attacks that result in packet dropouts or repetitions. However, it may not be effective in detecting replay attacks or stealthy data injection attacks, as these types of attacks do not cause deviations in the received data. To address this limitation, a dedicated attack detector can be incorporated into the method. This detector would assess whether the received data have been tampered with and discard any data that is deemed to be malicious. Despite this limitation, the proposed FD method can still improve the overall detection performance. This two-stage strategy offers a comprehensive security monitoring solution for NCSs. Various existing techniques can be utilized for the attack detection stage, such as [15,25]. The integration of these attack detectors with the proposed parity space-based FD method shows promise for future research.

## 5. Conclusions

In this paper, the FD problem in NCSs subject to the DoS attacks is addressed using a parity space-based approach. The proposed method utilizes all available input and output data for FD unit within a time-varying window to establish a parity relation. A novel residual generation scheme is presented, without introducing the transmission errors caused by DoS attacks in the sensor-to-FD unit. Furthermore, an adaptive residual evaluation function, along with a unified threshold is developed for different scenarios. This helps maintain a low FAR, even in the presence of DoS attacks that may cause packet dropouts. An online FD scheme is also proposed, with separate residual generation and evaluation schemes for scenarios with and without DoS attacks. Simulations on a linearized longitudinal model of an AUV have been used to demonstrate the effectiveness of the approach. The results show that the proposed approach achieves a lower FAR and higher F1 value compared to existing parity space-based FD schemes and KF-based FD schemes under DoS attacks, while maintaining a high FDR.

However, it is important to acknowledge the limitations of this study. The proposed method is specifically designed for LTI systems with DoS attacks that disrupt data availability. It is not applicable to replay attacks or stealthy data injection attacks, where the received data are complete but maliciously made. To address these types of attacks, it may be beneficial to extend the parity space framework by incorporating time-stamping or cryptographic techniques. Additionally, it should be noted that the current work only considers LTI dynamics and does not account for nonlinear or time-varying systems. To fully understand the effectiveness of the proposed approach, it will be necessary to investigate its performance on these types of systems in future studies.

## Figures and Tables

**Figure 2:**
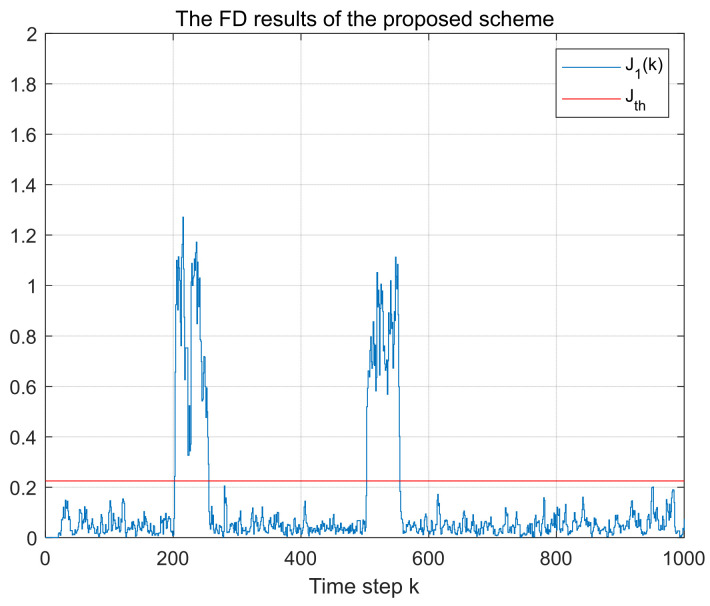
The FD results using the proposed scheme for Case 1.

**Figure 3:**
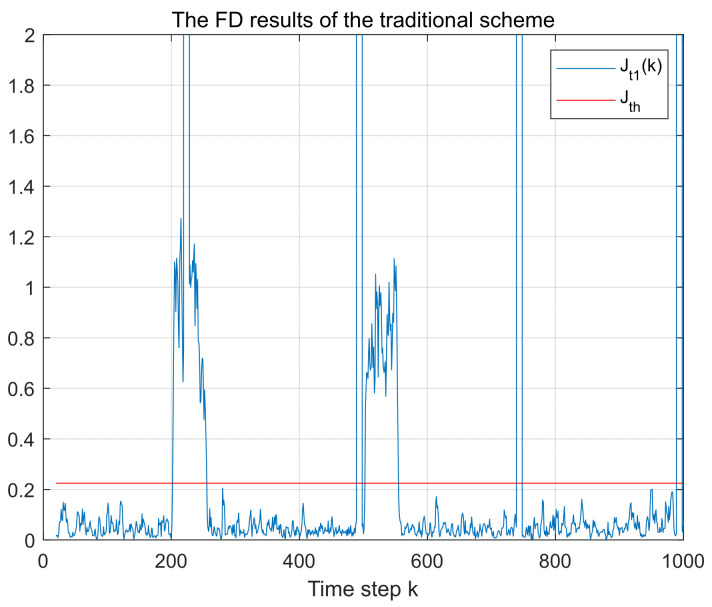
The FD results using the traditional scheme for Case 1.

**Figure 4:**
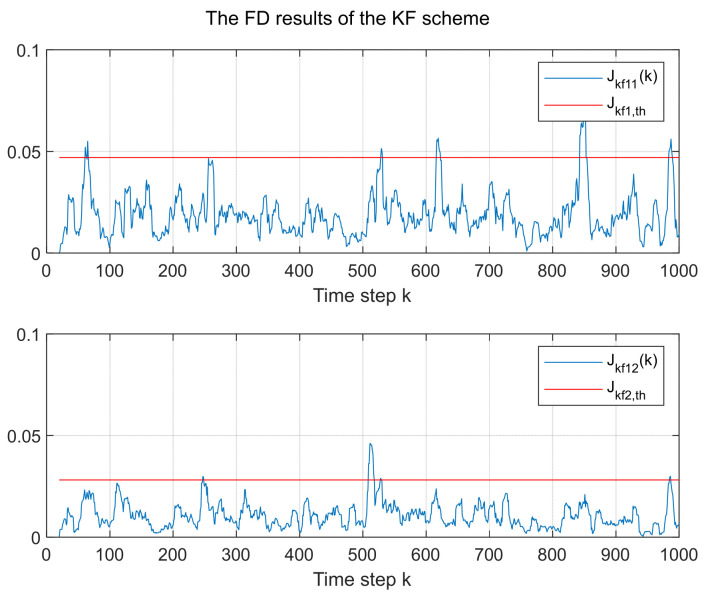
The FD results using the KF scheme for Case 1.

**Figure 5:**
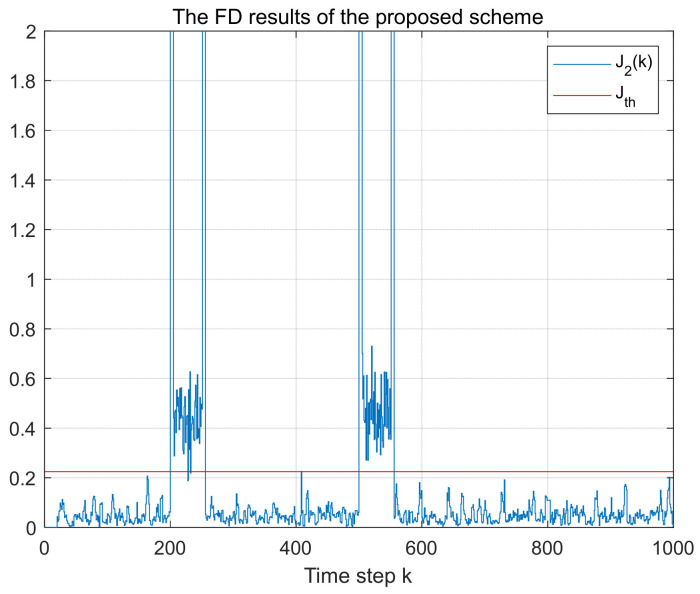
The FD results using the proposed scheme for Case 2.

**Figure 6:**
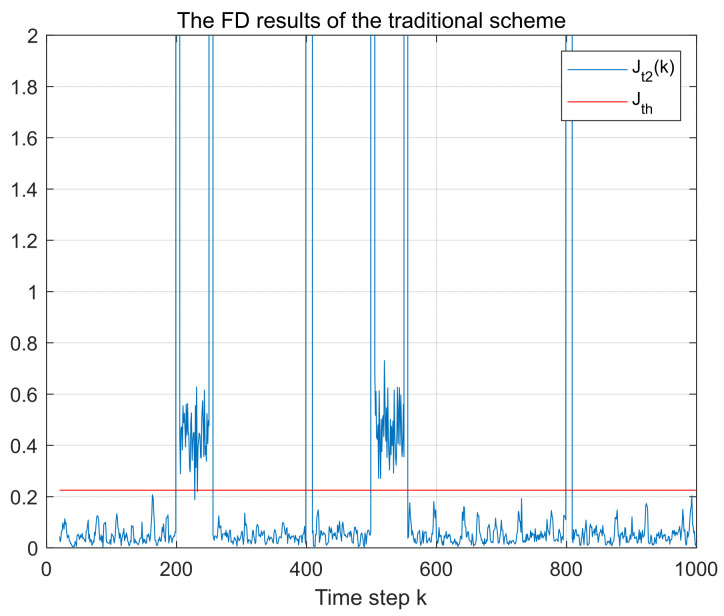
The FD results using the traditional scheme for Case 2.

**Figure 7:**
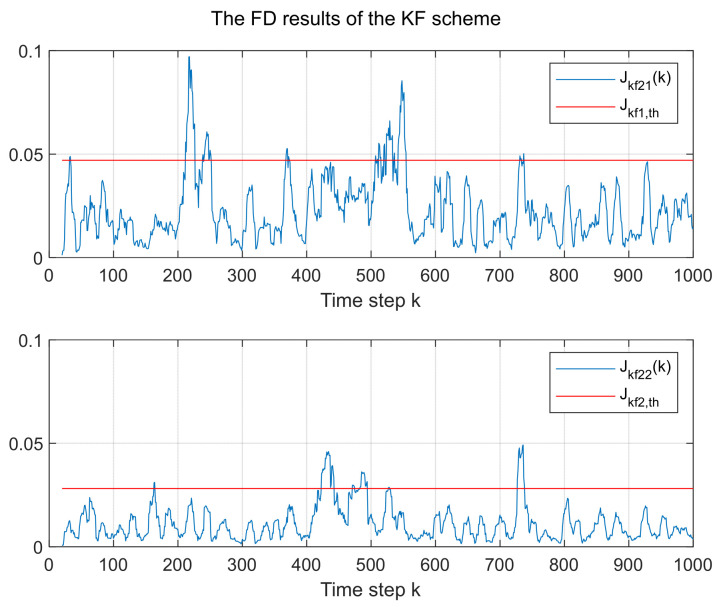
The FD results using the KF scheme for Case 2.

**Figure 8:**
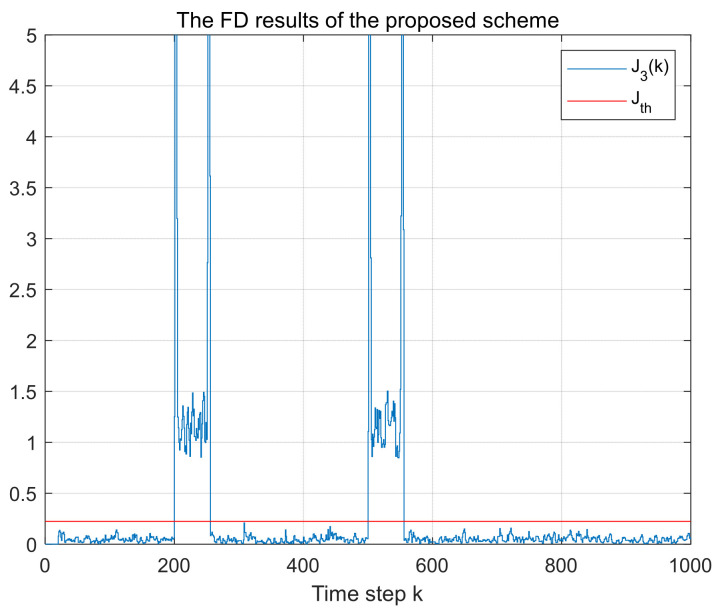
The FD results using the proposed scheme for Case 3.

**Figure 9:**
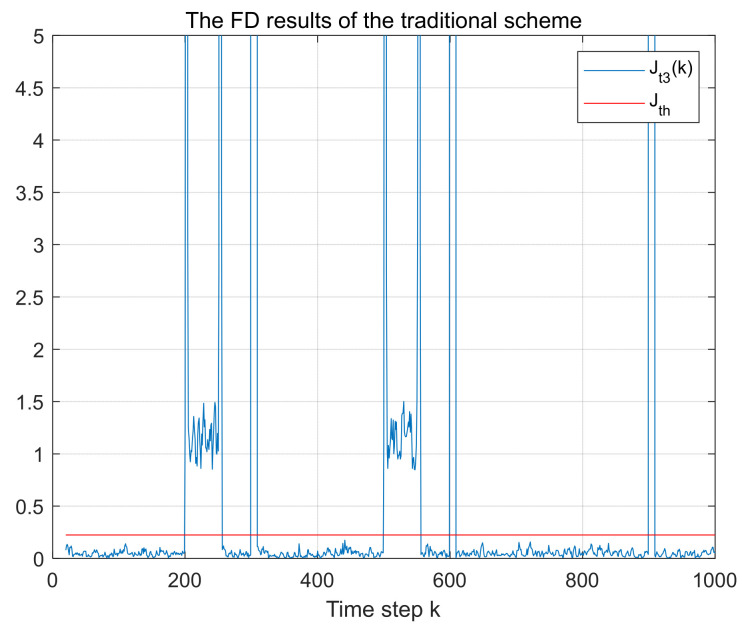
The FD results using the traditional scheme for Case 3.

**Figure 10:**
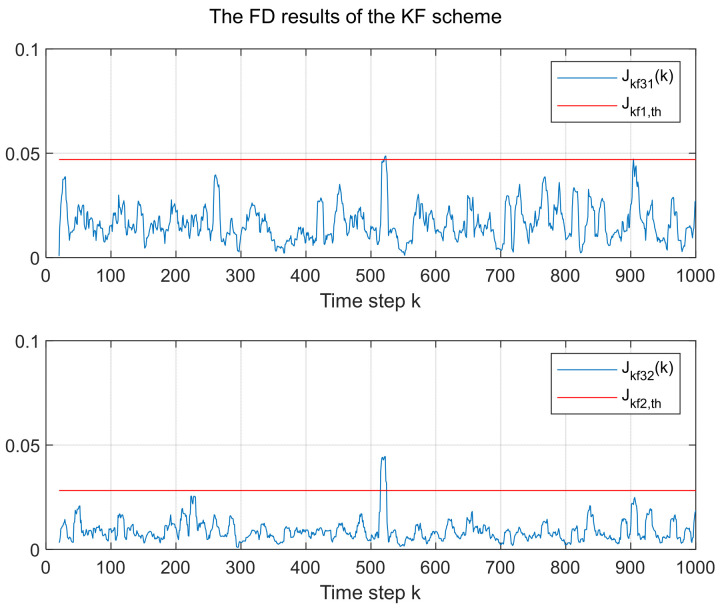
The FD results using the KF scheme for Case 3.

**Table 1:** A brief comparison of the three FD methods under DoS attacks.

Aspects	The Proposed Scheme	Traditional Parity Space Scheme	KF-Based Scheme
FD Method under the DoS attacks	Parity relations with adaptive time-varying	Parity relations with fixed windows + ZOH	Observer-based scheme with ZOH
Handling of missing data	Uses only available data, no substitution	Substitutes missing data with last value	Substitutes missing data with last value
Transmission errors in residual	Eliminated	Present (indistinguishable from faults)	Present (affecting the innovation)
Threshold Setting	Rigorous from bounds of unknown input	Heuristic (trial-and-error)	Statistical
Guaranteed FAR	Yes (theoretically zero)	No (affected by DoS attacks)	No (pre-tuned, increases under attacks)

**Table 2:** Performance metrics under DoS attacks (average values over Cases 1–3).

Performance Metrics	The Proposed Scheme	Traditional Parity Space Scheme	KF-Based Scheme
FDR/Recall	98.37%	98.37%	24.84%
MDR	1.63%	1.63%	75.16%
FAR	0.91%	3.64%	2.62%
Precision	92.62%	74.00%	52.41%
F1-score	95.41%	85.46%	33.71%
Detection Delay	0.5 sampling steps	0.5 sampling steps	/

## Data Availability

The data presented in this study were generated through simulations. No experimental data were collected. The simulation models and code are available from the corresponding author on reasonable request.
